# Self-training in significance space of support vectors for imbalanced biomedical event data

**DOI:** 10.1186/1471-2105-16-S7-S6

**Published:** 2015-04-23

**Authors:** Tsendsuren Munkhdalai, Oyun-Erdene Namsrai, Keun Ho Ryu

**Affiliations:** 1 Database/Bioinformatics Laboratory, Chungbuk National University, Cheongju, South Korea; 2 School of Information Technology, National University of Mongolia, Ulaanbaatar, Mongolia

## Abstract

**Background:**

Pairwise relationships extracted from biomedical literature are insufficient in formulating biomolecular interactions. Extraction of complex relations (namely, biomedical events) has become the main focus of the text-mining community. However, there are two critical issues that are seldom dealt with by existing systems. First, an annotated corpus for training a prediction model is highly imbalanced. Second, supervised models trained on only a single annotated corpus can limit system performance. Fortunately, there is a large pool of unlabeled data containing much of the domain background that one can exploit.

**Results:**

In this study, we develop a new semi-supervised learning method to address the issues outlined above. The proposed algorithm efficiently exploits the unlabeled data to leverage system performance. We furthermore extend our algorithm to a two-phase learning framework. The first phase balances the training data for initial model induction. The second phase incorporates domain knowledge into the event extraction model. The effectiveness of our method is evaluated on the Genia event extraction corpus and a PubMed document pool. Our method can identify a small subset of the majority class, which is sufficient for building a well-generalized prediction model. It outperforms the traditional self-training algorithm in terms of f-measure. Our model, based on the training data and the unlabeled data pool, achieves comparable performance to the state-of-the-art systems that are trained on a larger annotated set consisting of training and evaluation data.

## Background

As biomedical literature on servers grows exponentially in the form of semi-structured documents, biomedical text mining has been intensively investigated in order to find information in a more accurate and efficient manner. The previous efforts have focused on recognition of entity mentions, such as genes, proteins, diseases, or drug names [1-6], and on extraction of pairwise relationships, such as protein-protein interaction [7] and gene-disease association [8]. The named entities recognized, and pairwise relationships extracted, are insufficient for understanding biomolecular interactions [9]. Therefore, extraction of complex relations (namely, biomedical events) has received increasing attention. According to the BioNLP series challenges [10-12], a biomedical event is formulated as follows: an event has a trigger, a type and a set of arguments. An element of the argument set has a role and can be a protein mention or another event, depending on the event type. For example, 'RFLAT-1 activates RANTES gene expression' (PMID: 10023774) describes two events, one is simple gene expression of RANTES and the other is complex positive-regulation event which is caused by RFLAT-1, anchored by 'activates' and has the gene expression event as its argument. The BioNLP GE'09 and '11 challenges target three subtasks addressing event extraction at different levels of specificity: event detection, event enrichment and negation, and speculation detection. As solutions to the BioNLP challenges, many methods have been proposed to predict biomedical events from text. The solutions include rule-based and machine learning (ML) approaches. Rule-based event extraction systems rely on a rule set that is manually collected or automatically induced from training data [13-16]. The rule-based systems tend to achieve high precision with low recall and to perform better on prediction of simple events. To increase the recall, a rule-generation process has to process a huge amount of text to collect the rule set with high coverage. Since most computation is for matching pre-generated rules against text, such systems show good performance in terms of computation efficiency.

An ML-based event extraction system sees the task as a classification problem. The proposed approaches can be divided into three groups, depending on how recognition of the event trigger and argument is designed. The systems belonging to the first group are based on a text-mining pipeline approach [17-20]. Björne *et al*. [17] first adopted the pipeline approach in their Turku event extraction system. The first version of the Turku system consists of three stages: trigger detection, edge detection, and event duplication. The first two stages solve ML classification problems, and the last relies on a rule set. Miwa *et al*. [18] later improve the pipeline approach in their EventMine system by introducing an additional classification model for the rule-based event duplication task. In general, each stage in the extraction pipeline solves multi-class and multi-label classification problems based on an imbalanced dataset with a high dimensional feature space. A linear support vector machine (SVM) with one-versus-rest label decoding has been a main tool for this. For this group of approaches, errors made in a former step propagate into subsequent steps, introducing an error cascade. To overcome this issue, the second ML-based group uses global models that solve the whole task at once [21,22]. Riedel *et al*. [21] encoded graph structures of events using a set of binary variables representing the type of token nodes and the relations between them. The state of these variables is predicted by maximizing the global likelihood of integer linear programming. This joint model achieves good performance, but could be overly complicated for finding optimal states because it has to include every combination of tokens in the search space. To reduce the search space, they use a dictionary of triggers from the training data. This might in turn decrease the overall recall. McClosky *et al*. [22] aimed at solving the task as dependency parsing by exploiting global properties of event structures. The third ML-based group combines the global and pipeline approaches by using a pairwise model [23]. The pairwise model jointly predicts the trigger and the argument of an event as a pair of text parts, in contrast to the pipeline approach. However, such a model still has subsequent steps for prediction of events with more than one argument, and is unable to extract nested events.

There are two critical issues that are seldom dealt with by the aforementioned systems. First, training data is highly imbalanced. From traditional sampling [24] (under-sampling or over-sampling) to active learning [25], solutions have been tooled to induce prediction models on such imbalanced datasets [26]. In Björne *et al*. [27] and the EventMine system, a simple class weighting method with an SVM [28] is used. Second, the supervised models trained on only a single annotated corpus can limit system coverage and scalability. Besides merging multiple annotated corpora into one [29], semi-supervised learning (SSL) is applied to overcome this issue [30]. SSL has received significant attention for two reasons. First, annotating data for training is time- and labor-intensive. For instance, annotating the Genia event corpus consisting of 9372 sentences required 1.5 years with five part-time annotators and two coordinators [31]. Second, because SSL exploits unlabeled data, the accuracy of classifiers is generally improved.

In this study, we combine the approaches of active learning and self-training and develop a new semi-supervised learning method to address the issues outlined above. Our algorithm is built upon the foundation of significance space construction [32]. The training data are augmented by a new example set from unlabeled data, so model learning with them captures patterns from domain background. The new example set is formed based on its significance and confidence score for self-labelling.

Furthermore, we extend the algorithm to a two-phase learning framework. The first phase balances the training data for initial model induction, whereas the second phase incorporates domain knowledge into the model by querying the example set from the unlabeled data. In both phases, the model is built in an online fashion. We evaluated the proposed method on the GE'11 corpus. First, we compared the method against the approaches used to solve the data imbalance problem. Our method can identify a small subset of the majority class that is sufficient for building a well-generalized prediction model. Second, we contrasted it against the traditional self-training algorithm as an evaluation of the semi-supervised learning perspective. Finally, we investigated the event extraction system performance, relying on our proposed method to report the different values of the evaluation measures along with the GE'11 shared task entries. Our model, which learned only on the training data, achieves comparable performance to the state-of-the-art systems that are trained on both GE evaluation and training data.

## Methods

We combine the approaches of active learning and self-training to develop a new semi-supervised learning method, which we call self-training in significance space (STSS). An STSS-based two-phase learning framework is also proposed in this study, in order to leverage system performance and to solve the data imbalance issue by exploiting unlabeled data.

### Text preprocessing and feature extraction

Text preprocessing where text data is cleaned and processed via natural language processing (NLP) tools is a preparatory task for the feature extraction step. We adopt the Turku system [27] for preprocessing and feature extraction. Turku allows extracting a rich number of features, and is well tuned for processing PubMed abstracts for the purpose of event extraction.

In preprocessing, the text data is cleaned by removing non-standard characters, and is then processed with NLP tools to have its sentences split and parts of speech (POS) tagged and parsed. We use the Genia Sentence Splitter (GeniaSS) [33] and the Charniak-Johnson parser [34] with McClosky's biomedical parsing model [35]. The GeniaSS relies on the maximum entropy models and is optimized for bio text data. McClosky's model was built with self-training, incorporating the domain knowledge from PubMed abstracts.

In addition to the preprocessing step, we recognize biomedical named entities in the unlabeled text. We use the Bayesian finite state model (BFSM) with the Bayesian classifier from our previous study [36] and the BANNER tool [37] for biomedical named entity recognition, in order to improve recognition accuracy. Our named entity recognition system solves the name boundary issue and achieves a high precision, whereas the BANNER tool utilizes a sequence labeling model, conditional random fields, and shows reliable performance on the task.

Once a graph representation of the full dependency within a sentence is obtained, we extract features for the event extraction model with the Turku system. The following four different feature sets are extracted.

*Token features: *orthographic features, POS tags, base words with the Porter stemmer, and character n-grams (n = {1, 2, 3})

*Sentence features: *the number of entities and bag-of-words

*Sentence dependency features: *n-grams of the words on the shortest path between two entities, and features based on triggers present

*External resource features: *WordNet hypernyms [38], and a similarity measure against lexicons of biomedical terms.

### Self-training in significance space of support vectors

**Figure 1:**
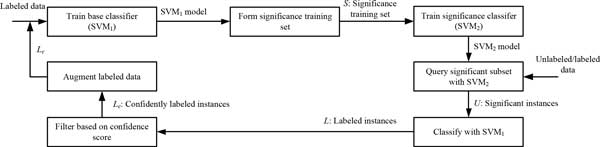
**The flow chart of the STSS method**.

The proposed semi-supervised learning method relies on the concept of significance space, which could be constructed by the different approaches from the original training data. Significance space with an SVM classifier depends on the feature space of the support vectors (SVs). The flow chart of the proposed method is shown in Figure 1.

First, the original training set is used to build the base classifier, the **SVM_1 _**model. STSS then forms the significance training set, **S**, by labeling the training examples that are SVs of **SVM_1 _**for significance and the remaining examples of the training set for non-significance. In the next step of the current round, the **SVM_2 _**model is trained via **S **in order to query the significant subset, **U**, from unlabeled or labeled data. Different usages of STSS in the learning framework are discussed in the next section The **SVM_2 _**model can be applied to either labeled data, to select most informative labeled examples, or to unlabeled data, to let the **SVM_1 _**model give class labels to the informative subset. If the SVM model classifies the significance examples of the unlabeled data, then the confidence-based filtering module is employed to select the confidently labeled examples, **L_c_**, with a threshold criterion. We investigate various instance selection strategies, most of which are based on the probability outcome from the classification model:

1. Top-k example, e.g. the labeled instances are ranked according to the confidence score, and the top-k number of examples from the top are added to the training set

2. Top-k percentage, e.g. the labeled instances are ranked according to confidence score, and the k percentage of all instances from the top are selected

3. Examples satisfying a confidence threshold, e.g. a set of examples where probability outcomes for a particular class are higher than a threshold

4. Randomly selected examples

The first and third strategies can be seen as a hard threshold, whereas the second strategy defines flexible thresholds because the number of examples to be selected depends on the total number of labeled examples at the moment.

### STSS-based two-phase learning framework

We propose a two-phase learning framework based on the STSS algorithm to solve the imbalanced data problem, while exploiting unlabeled data efficiently. The first phase solves the data imbalance problem by selecting only a small and informative subset of the majority class. First the original training data, **D**, is partitioned into two parts: **D_i _**to be initial training data, and **D_u _**to be used as the unlabeled data (**D_i _**<<**D_u_**) for the STSS algorithm. Then, the STSS runs to build the **SVM_1 _**model while drawing a balanced subset of the original training data: **D_s_**. Since each instance in the **D_u _**set already has a true class label, the self-labeling schema with **SVM_1 _**is not utilized for the first run of the STSS (the STSS run of Phase I).

The second phase is designed to exploit unlabeled data in order to improve the performance of the **SVM_1 _**model outputted by the previous stage. The **SVM_1 _**model, a balanced subset of training data **D_s _**and previously prepared unlabeled data **U**, is the input for this phase. We simply run the STSS and update the **SVM_1 _**model with the self-labeled informative examples chosen from the unlabeled data. For each running iteration of the STSS, the **SVM_1 _**model is updated and stored for further evaluation.

### Base classifier

The dataset extracted from a biomedical event corpus simply becomes high-dimensional, having hundreds of thousands of attributes, and needs a solution to the multi-label and multi-class classification problem. An SVM classifier with a linear kernel is a benchmark in classifying such high-dimensional data.

The linear SVM classifier is well suited to a binary classification problem, and thus requires additional wrapper methods to deal with the multi-label and multi-class classification task. We use a one-versus-rest approach with linear SVM. The one-versus-rest approach builds the same number of classifiers as the classes in the dataset, treating each classifier as an individual component to discriminate between the instances of one class and the instances of other classes, and combines the classifiers in a simple voting schema to make the class decision on a test instance. In the experiment, we train the classifiers in a parallel manner, running a per-core learning process to speed up the model induction process.

## Results

We evaluated the proposed method on the GE'11 dataset by comparing its performance with state-of-the-art systems. First, we compared the method against the approaches used to solve the data imbalance problem. Second, in order to observe the effectiveness of the method from the semi-supervised learning perspective, we contrasted it against the traditional self-training algorithm. These comparisons were accomplished by using a dataset generated for the edge detection subtask. Finally, we investigated the event extraction system performance, relying on our proposed method to report the different values of the evaluation measures along with the GE'11 shared task entries. Our source code for the algorithm, the two-phase learning framework, and the other benchmarks are available at https://bitbucket.org/tsendeemts/stss.

### Text corpus and dataset

Because the STSS method is a semi-supervised learning method and is able to exploit raw text data, we prepared a PubMed document collection in addition to the GE'11 dataset. Therefore, we used the GE'11 dataset as labeled data and the PubMed collection consisting of around 1,400,000 abstracts as unlabeled data for this experiment. The GE'11 dataset is a super-set of the GE'09 dataset [11] and is split into training, development and testing sets, consisting of 800 abstracts (+5 full papers), 150 abstracts (+5 full papers), and 260 abstracts (+4 full papers), respectively. We applied the training set for model induction and the development set for evaluation of models for the subtasks throughout the experiment in this study. Finally, the test set was used to report the whole-system performance based on the STSS method.

We adopted the pipeline approach, so the three different datasets (for trigger detection, edge detection or event construction) were extracted from the original set. Table 1 provides the class label distribution over the instances in the training set for edge detection. The first column and the second column contain the class label representing trigger-entity connection and the number of instances belonging to every class, respectively. The third column reports the class imbalance ratio to demonstrate one of our main concerns in this study: that the event extraction datasets are highly imbalanced. In fact, event detection and trigger detection are considered to be challenging classification problems in machine learning, caused by the data imbalance nature and the high dimensionality. The trigger detection dataset extracted in this experiment consists of 19,614 positive examples (belonging to 31 different problem-specific classes) and 141,564 negative examples and has 412,753 features.

**Table 1:** Class distribution in the training set for edge detection.

Class label	Number of instances	Imbalance ratio
AtLoc	48	1:76282

Cause	1117	1:3277

Cause-Theme	6	1:610261

Site	425	1:8614

Site-Theme	3	1:1220524

Theme	9246	1:395

ToLoc	50	1:73231

Negative	3650680	1:0.002

### STSS for the imbalanced data problem

We compared the performance of the STSS method against the baseline approaches, under-sampling and the class weighting methods on the edge detection dataset. The class weighting method is tightly integrated with SVM classifiers and has been used previously in many event extraction systems [17,18,23].

Since there are only three instances of the Site-Theme class in the training dataset, we removed this class and reduced the number of distinct class labels to 7. The different settings for each baseline were evaluated on development to tune the corresponding hyper-parameters, and the best setting giving the highest accuracy was selected. We under-sampled the negative examples so that the class distribution remained in ratios of 1/3, 1/4, 1/5 and 1/6, and the best sampling reported in this experiment was the dataset with a ratio of 1/4. For the class weighting run, every class received a weight that is inversely proportional to the class frequency; therefore, the minor classes received a higher score than the major classes. The second strategy among the instance selection strategies defined in Section 2.2 worked slightly better than the others in our STSS implementation.

The overall performance of the different approaches is shown in Table 2. As we expected, STSS performed best on this imbalanced dataset, followed by the class weighting method. Surprisingly, none of the methods were able to classify instances of the AtLoc class, showing 0.0% for the f-measure. This might be due to non-representative features for the class. We used the SVM classifier with a linear kernel that is able to train on the dataset with a large number of attributes, even though arbitrary classifiers could be applied to the under-sampled and class-weighted datasets.

**Table 2:** Comparison of solutions to the data imbalance issue on the edge detection dataset.

Class label	Precision (%)			Recall (%)			F-measure (%)		
	**Under-sampling**	**Class weighting**	**STSS**	**Under-sampling**	**Class weighting**	**STSS**	**Under-sampling**	**Class weighting**	**STSS**

AtLoc	0.0	0.0	0.0	0.0	0.0	0.0	0.0	0.0	0.0

Cause	100.0	42.24	47.36	4.48	25.95	43.67	8.51	32.14	**45.44**

Cause-Theme	100.0	63.07	76.4	40.47	63.32	50.21	57.62	**63.19**	60.59

Site	0.0	20.78	29.3	0.0	12.37	33.21	0.0	15.5	**31.13**

Theme	53.87	60.91	57.16	42.21	62.37	90.83	47.33	61.63	**70.16**

ToLoc	26.91	57.39	48.2	99.74	54.33	96.11	42.38	55.81	**64.2**

Negative	89.66	92.35	100.0	100.0	100.0	100.0	94.54	96.02	**100.0**

Finally, we investigated the data efficiency of STSS by comparing original and STSS-training datasets constructed especially for one-versus-rest SVM model induction.

The STSS training dataset is an informative subset of the original, effectively sampled through the first phase of the STSS-based two-phase learning. We started the learning process by feeding all the positive examples with the same number of randomly chosen negative examples, and STSS proceeds further by sampling an informative set of the negative examples in every iteration.

The comparison is shown in Table 3 which reports the statistics for the original and the STSS training datasets. Sampling the informative subset dramatically increases the imbalance ratio (an imbalance ratio close to 1.0 is preferred). We can see that the only a small subset of the negative examples in the original dataset can represent the others, and our STSS method can identify them effectively. For example, for the Cause class, STSS sampled 27,505 negative examples out of the 3,660,458 examples.

**Table 3:** Data efficiency of the STSS algorithm.

Class label	Original training dataset		STSS training dataset (Ds)	
	**Number of instances**	**Imbalance ratio**	**Number of instances**	**Imbalance ratio**

AtLoc	Pos: 48 Neg: 3661527	1:76282	Pos: 48 Neg: 128761	1:2682

Cause	Pos: 1117 Neg: 366045	1:3277	Pos: 1117 Neg: 27505	1:24

Cause-Theme	Pos: 6 Neg: 3661521	1:610261	Pos: 6 Neg: 6000	1:1000

Site	Pos: 425 Neg: 36661150	1:8614	Pos: 425 Neg: 36627	1:86

Theme	Pos: 9246 Neg: 3652329	1:395	Pos: 9246 Neg: 30915	1:3

ToLoc	Pos: 50 Neg: 3661525	1:73231	Pos: 50 Neg: 167120	1:3342

An imbalance ratio close to 1.0 is preferred.

### Comparison with traditional self-training

In the second phase of the STSS-based learning framework, the STSS algorithm was employed on both labeled and unlabeled data, acting as a typical semi-supervised learning method. Since STSS could be viewed as a variation of self-training, we compared our method with traditional self-training.

We ran the methods five times by using the edge detection training dataset for training and the development dataset for evaluation, and reported the best performance of both methods in contrast.

The overall performance of the two methods is shown in Table 4. We see that our method achieves a higher recall value without losing precision, improving the f-measure in general. The STSS algorithm outperforms self-training by 14.18% for f-measure in the Cause-Theme class. Since the dataset is highly imbalanced, general measurements like precision, recall, and f-measure reported a value of 100% for the negative class.

**Table 4:** Comparison of the STSS algorithm with traditional self-training on the edge detection dataset.

Class label	Precision (%)		Precision (%)		F-measure (%)	
	**Self-training**	**STSS**	**Self-training**	**STSS**	**Self-training**	**STSS**

AtLoc	0.0	0.0	0.0	0.0	0.0	0.0

Cause	47.36	49.21	43.54	43.61	45.36	**46.24**

Cause-Theme	76.2	67.85	53.72	80.6	63.01	**73.67**

Site	29.82	29.25	33.2	33.0	**31.4**	31.01

Theme	57.11	57.72	90.33	90.92	69.97	**70.61**

ToLoc	48.42	48.94	96.42	96.68	64.46	**64.98**

Negative	100.0	100.0	100.0	100.0	100.0	100.0

### Performance Evaluation

In this section, we evaluated the performance of the event extraction system based on the proposed method and compared it with GE'11 entries. We trained the classification models of the system with the GE'11 training corpus and then tested the system performance with the GE'11 test corpus (Task 1).

Table 5 shows the results of the extraction method evaluated on the test dataset using the Approximate Span/Approximate Recursive matching criteria. The evaluation results of the abstract and full text documents are separately reported to show the type of document on which our method performs better. Our method tends to perform better on full-text documents. This can also be seen from Table 6 where we compare the evaluation results with other GE'11 entries. Our results in terms of f-measure are slightly higher than Turku's result on the full-text documents.

**Table 5:** Evaluation results of the test dataset.

Event class	Abstract			Full text		
	**R**	**P**	**F**	**R**	**P**	**F**

Gene_expression	73.68	78.68	75.78	81.43	74.51	77.82

Transcription	53.28	65.18	58.63	35.14	56.52	43.33

Protein_catabolism	42.86	85.71	57.14	100.00	100.00	100.00

Phosphorylation	82.96	79.43	81.16	90.00	93.75	91.84

Localization	25.86	75.00	38.46	82.35	70.00	75.68

SVT-TOTAL	64.97	76.65	70.33	78.18	75.63	76.88

Binding	45.24	49.84	47.43	37.50	31.58	34.29

EVT-TOTAL	60.50	70.24	65.00	67.11	62.39	64.66

Regulation	29.55	41.95	34.68	27.66	34.21	30.59

Pos_regulation	42.12	50.49	45.92	38.04	48.61	42.68

Neg_regulation	44.33	48.98	46.54	31.25	40.54	35.29

REG-TOTAL	40.41	48.83	44.22	34.99	44.69	39.25

ALL-TOTAL	50.06	59.33	54.30	48.31	53.43	50.74

P: Precision, R: Recall, F: F-measure.

**Table 6:** Performance comparison with GE'11 entries.

System	Abstract			Full text		
	**R**	**P**	**F**	**R**	**P**	**F**

UMass[21]	48.74	65.94	56.05	47.84	59.76	53.14

Turku [27]	50.06	59.48	54.37	48.31	53.38	50.72

MSR-NLP [20]	48.52	56.47	52.20	44.71	57.57	50.40

Concord U [13]	43.09	60.37	50.28	48.94	50.77	49.84

Ours	50.06	59.33	54.30	48.31	53.43	50.74

P: Precision, R: Recall, F: F-measure.

The overall evaluation results are closer to that of Turku because we injected the classification models trained using STSS into Turku's pipeline, sharing the same preprocessing and feature extraction stages. However, our STSS model uses the training dataset and the unlabeled data, whereas the Turku model uses a larger annotated set consisting of the training and evaluation datasets.

### Event extraction-based applications

Event extraction from biomedical text benefits a broad range of applications in systems biology, namely literature searching, interaction network generation and pathway construction. Biomedical events extracted from literature are indexed to support a deeper semantic search in comparison to traditional keyword-based search engines. Thus, such a search system can provide results with a high precision to a user. BioContext [39] and MEDIE [40] systems are example of event-based search engines. MEDIE has an intelligent interface for retrieving biomedical events referenced with their literature from the entire MEDLINE. In MEDIA, the user can even use a partial query to have a rich number of results and browse through them. Like MEDIE, BioContext integrates a several different text mining tools, including named entity recognition, entity normalization, full dependency parsing and event extraction to build up. BioContext system processed 10.9 million MEDLINE abstracts and 234 000 full-text articles and made 11.4 million distinct events searchable.

One of the applications based on event extraction is the generation of interaction network. Sufficient accurate event graphs can be used for inferring complex regulatory relationship networks and other biologically relevant tasks [27]. Björne *et al*. [27] applied their Turku system to 1% of MEDLINE and constructed connected components of the event graph. While there is a wide range of application areas where the event extraction can be useful, many issues remained to be solved in practice. The system performance is still lower in terms of F-measure. The best performing system for GE'11 challenge task showed 53.14% F-measure. Another issue to be addressed is computational requirements. ML-based systems require a large computation time, mostly devoted by full dependency parser in preprocessing step. Björne *et al*. [27] reported that their Turku system took 98 processor hours (411 processor days for the entire MEDLINE) to extracts events from only 1% of MEDLINE. Even using a cluster of 100 processors, BioContext required 2-3 months to process the full document collection [39]. We used a cluster of 12 processor cores, 4 of which for ML example construction from unlabeled data and the others for training of SVM models. It took ~3 months to complete the experiments.

## Conclusions

In this study, we proposed a new semi-supervised method and its two-phase framework to build a biomedical event extraction model with imbalanced data. Our method iteratively constructs significance space from training data and augments it with a self-labeled example set that falls into that space. Based on this online process, our learning framework in its first phase forms a balanced subset of the original data for initial model induction, and then in the second phase, incorporates domain knowledge from unlabeled data into the model. Consequently, the framework not only solves the data imbalance problem, but also exploits the unlabeled data to leverage system performance.

Experimental results demonstrated the efficiency of our method from multiple perspectives. Our method can identify a small and sufficient subset of the majority class. It outperforms the traditional self-training algorithm in terms of f-measure. Our method builds a well-generalized prediction model with a small training set and additional unlabeled data. The proposed method can be applied to other real-world applications where training data might be small and imbalanced, and unlabeled data is less expensive to collect.

For future work, we will explore representation learning to integrate external resources into biomedical event extraction. We will also investigate methods to avoid complex preprocessing (e.g. full dependency parsing) and to address error cascading. Deep learning methods might help in this.

## Competing interests

The authors declare that they have no competing interests.

## Authors' contributions

TM conceived the study, participated in its design, developed the benchmark programs, and drafted the manuscript. ON helped draft the manuscript. KHR provided system design, valuable guidance, editing, and research grant. All authors read and approved the final manuscript.

## Declarations

The publication costs for this article were funded by the Basic Science Research Program through the National Research Foundation of Korea (NRF) funded by the Ministry of Science, ICT and Future Planning (No-2013R1A2A2A01068923) and by a National Research Foundation of Korea (NRF) grant funded by the Korea government (MSIP) (No. 2008-0062611).

This article has been published as part of *BMC Bioinformatics *Volume 16 Supplement 7, 2015: Selected articles from The 11th Annual Biotechnology and Bioinformatics Symposium (BIOT-2014): Bioinformatics. The full contents of the supplement are available online at http://www.biomedcentral.com/bmcbioinformatics/supplements/16/S7.
